# {*N*,*N*-Bis[2-(trimethyl­silylamino)eth­yl]-*N*′-(trimethyl­silyl)ethane-1,2-diamin­ato(3–)-κ^4^ *N*}methyl­zirconium(IV)

**DOI:** 10.1107/S160053680800425X

**Published:** 2008-02-15

**Authors:** Samantha N. MacMillan, Joseph M. Tanski, Rory Waterman

**Affiliations:** a Department of Chemistry, Vassar College, Poughkeepsie, NY 12604, USA; b Department of Chemistry, University of Vermont, Burlington, VT 05405, USA

## Abstract

The title compound, [Zr(CH_3_)(C_15_H_39_N_4_Si_3_)], is a unique example of a triamido­amine-supported zirconium–methyl complex that crystallized as a monomer with trigonal–bipyramidal geometry at zirconium, featuring a Zr—C bond of 2.2963 (16) Å.

## Related literature

For recent applications of (N_3_N)ZrMe in catalysis, see: Waterman (2007 ▶); Roering *et al.* (2007 ▶, 2008 ▶). For examples of structurally characterized triamido­amine-supported zirconium complexes, see: Duan *et al.* (1995 ▶); Morton *et al.* (1999 ▶, 2000 ▶); MacMillan *et al.* (2007 ▶). For related literature, see: Addison *et al.* (1984 ▶); Parkin (1992 ▶).
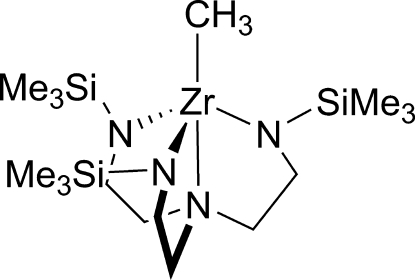


## Experimental

### 

#### Crystal data

[Zr(CH_3_)(C_15_H_39_N_4_Si_3_)]
*M* *_r_* = 466.03
Orthorhombic, 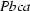

*a* = 15.6018 (7) Å
*b* = 18.0682 (8) Å
*c* = 18.3745 (8) Å
*V* = 5179.7 (4) Å^3^
*Z* = 8
Mo *K*α radiation
μ = 0.57 mm^−1^
*T* = 125 (2) K
0.24 × 0.20 × 0.16 mm

#### Data collection

Bruker SMART CCD area-detector diffractometer
Absorption correction: multi-scan (*SADABS*; Bruker, 1999 ▶) *T*_min_ = 0.875, *T*_max_ = 0.914
68275 measured reflections
6973 independent reflections
5714 reflections with *I* > 2σ(*I*)
*R*_int_ = 0.033

#### Refinement

*R*[*F* ^2^ > 2σ(*F* ^2^)] = 0.025
*wR*(*F* ^2^) = 0.063
*S* = 1.04
6973 reflections
226 parameters
H-atom parameters constrained
Δρ_max_ = 0.37 e Å^−3^
Δρ_min_ = −0.29 e Å^−3^

### 

Data collection: *SMART* (Bruker, 2001 ▶); cell refinement: *SAINT-Plus* (Bruker, 1999 ▶); data reduction: *SAINT-Plus*; program(s) used to solve structure: *SHELXS97* (Sheldrick, 2008 ▶); program(s) used to refine structure: *SHELXL97* (Sheldrick, 2008 ▶); molecular graphics: *SHELXTL* (Sheldrick, 2008 ▶); software used to prepare material for publication: *SHELXTL*.

## Supplementary Material

Crystal structure: contains datablocks global, I. DOI: 10.1107/S160053680800425X/hg2378sup1.cif

Structure factors: contains datablocks I. DOI: 10.1107/S160053680800425X/hg2378Isup2.hkl

Additional supplementary materials:  crystallographic information; 3D view; checkCIF report

## Figures and Tables

**Fig. 1.**
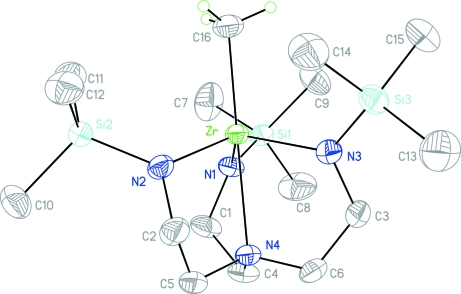
A view of complex I, with displacement ellipsoids shown at the 50% probability level. H atoms except those on C(16)have been omitted for clarity.

## supplementary crystallographic information

### Comment

Simple derivatives of triamidoamine-supported zirconium have recently been prepared, and these complexes have been found to be effective pre-catalysts for dehydrocoupling involving phosphines (Roering *et al.*, 2007; Waterman, 2007) and arsines (Roering *et al.*, 2008). Reaction of (N_3_N)ZrCl with MeLi in Et_2_O afforded (N_3_N)ZrMe (I, N_3_N = N(CH_2_CH_2_NSiMe_3_)_3_^3–^)) in 82% yield as colorless crystals (Waterman, 2007). Complex I is highly lipophilic and exhibits reasonable thermal stability despite a low melting temperature (Mp = 87–91°C). Upon heating in benzene solution, methane is evolved, and the triamidolamine ligand is metalated at the trimethylsilyl functionality—a key step in dehydrocoupling catalysis (Waterman, 2007). Whereas zirconium complexes with methyl ligands are commonplace, those supported by triamidoamine ligands were previously unknown.

Complex I was crystallized from cold pentane solution and exhibits distorted trigonal bipyramidal geometry at zirconium. The τ5-parameter for the complex was calculated as 1.1 (Addison *et al.*, 1984), though greater than 1, it is consistent with a trigonal bipyramidal geometry rather than square-pyramidal. The value greater than unity is attributed to the fact that the amido nitrogen atoms are not co-planar with Zr as expected for a trigonal pyramid, a feature that artificially increases the α angle in the τ5-parameter calculation.

The three amido ligands orient in a roughly *C*_3_-symmetric fashion about the Zr–C bonding axis with an average Zr—N bond length of 2.0714 (13) Å. The bond to the axial nitrogen appears to be effected by the relatively strong *trans*-directing methide ligand with Zr—N(4) = 2.538 (1) Å. This bond length is slightly longer than that observed for (N_3_N)Zr(PHPh) with Zr—N(4) = 2.526 (2) Å (Roering *et al.* 2007) and considerably longer than that seen for arsenido derivatives (N_3_N)ZrAsPh_2_, Zr—N(4) = 2.516 (2) Å, and (N_3_N)ZrAsHMes, Zr—N(4) = 2.502 (2) Å (Roering *et al.*, 2008).

For the methide ligand, the Zr—C bond length (2.2963 (16) Å) of I is slightly shorter than the most closely related complex structurally characterized complex (N_3_N*)ZrCH_2_Ph (N_3_N* = N(CH_2_CH_2_NSiMe_2_*^t^*Bu)_3_^3–^), which displays Zr—C 2.3243 (18) Å (Morton *et al.*, 1999). Of the *ca* 315 complexes featuring a Zr—CH_3_ bond, the Zr—C bond of I fits neatly into the range of reported Zr—C bond lengths of 2.046 to 2.805 Å.

The Hirshfeld Test Difference for Zr—C(16) is 6.71 su. This value is most likely the result of a small amount of contamination by the (N_3_N)ZrCl precursor. This is an interesting observation given the high analytical purity of I and the inability to observe any (N_3_N)ZrCl in benzene-*d*_6_ solutions of I by ^1^H NMR spectroscopy. However, residual electron density was observed along the Zr—C vector, but this peak could not be refined as a chloride disorder. Contamination of crystals by trace quantities of chloride derivatives is known, and due caution must be taken in evaluating data (Parkin, 1992). While this test does not significantly detract from the quality of this structure and solution and correlation between the structure and spectroscopic assignment, it does indicate that contamination of product complexes with (N_3_N)ZrCl is possible. This observation suggests that synthetic strategies for (N_3_N)ZrX derivatives that circumvent the use of (N_3_N)ZrCl are optimal for achieving highly pure compounds for catalytic applications.

### Experimental

Methyl complex (N_3_N)ZrMe (I) was prepared according to the literature procedure (Waterman, 2007). Dissolving complex I (*ca* 800 mg) in minimal pentane (2 ml), then filtering and cooling the resulting colorless solution to -30 °C for extended periods (*ca* 1–2 weeks) afforded colorless, X-ray quality crystals.

### Refinement

Hydrogen atoms on carbon were included in calculated positions and were refined using a riding model [C—H = 0.98 (CH_3_), 0.99 Å (CH_2_); *U*_iso_ = 1.5 *U*_eq_ (CH_3_), 1.2 *U*_eq_ (CH_2_)]. The Hirshfeld test difference value Zr—C(16) = 6.71 su indicates slight contamination with the chloro precursor. The slighly low *U*_eq_ value for Si(1) is likely the result of the terminal the atom residing in a trimethylsilyl substituent.

### Crystal data

**Table:**

[Zr(CH_3_)(C_15_H_39_N_4_Si_3_)]	*D*_x_ = 1.195 Mg m^−^^3^
*M**_r_* = 466.03	Melting point: 362 K
Orthorhombic, *P**b**c**a*	Mo *K*α radiation λ = 0.71073 Å
Hall symbol: -P 2ac 2ab	Cell parameters from 9832 reflections
*a* = 15.6018 (7) Å	θ = 2.5–30.2º
*b* = 18.0682 (8) Å	µ = 0.57 mm^−^^1^
*c* = 18.3745 (8) Å	*T* = 125 (2) K
*V* = 5179.7 (4) Å^3^	Block, colorless
*Z* = 8	0.24 × 0.20 × 0.16 mm
*F*_000_ = 1984	

### Data collection

**Table:**

Bruker SMART CCD area-detector diffractometer	6973 independent reflections
Radiation source: fine-focus sealed tube	5714 reflections with *I* > 2σ(*I*)
Monochromator: graphite	*R*_int_ = 0.033
*T* = 125(2) K	θ_max_ = 29.1º
φ and ω scans	θ_min_ = 2.1º
Absorption correction: multi-scan(SADABS; Bruker, 1999)	*h* = −21→21
*T*_min_ = 0.875, *T*_max_ = 0.914	*k* = −24→24
68275 measured reflections	*l* = −24→25

### Refinement

**Table:**

Refinement on *F*^2^	Secondary atom site location: difference Fourier map
Least-squares matrix: full	Hydrogen site location: inferred from neighbouring sites
*R*[*F*^2^ > 2σ(*F*^2^)] = 0.025	H-atom parameters constrained
*wR*(*F*^2^) = 0.063	*w* = 1/[σ^2^(*F*_o_^2^) + (0.0241*P*)^2^ + 2.7021*P*] where *P* = (*F*_o_^2^ + 2*F*_c_^2^)/3
*S* = 1.04	(Δ/σ)_max_ = 0.002
6973 reflections	Δρ_max_ = 0.37 e Å^−^^3^
226 parameters	Δρ_min_ = −0.29 e Å^−^^3^
Primary atom site location: structure-invariant direct methods	Extinction correction: none

### Special details

**Table:**

Geometry. All e.s.d.'s (except the e.s.d. in the dihedral angle between two l.s. planes) are estimated using the full covariance matrix. The cell e.s.d.'s are taken into account individually in the estimation of e.s.d.'s in distances, angles and torsion angles; correlations between e.s.d.'s in cell parameters are only used when they are defined by crystal symmetry. An approximate (isotropic) treatment of cell e.s.d.'s is used for estimating e.s.d.'s involving l.s. planes.
Refinement. Refinement of *F*^2^ against ALL reflections. The weighted *R*-factor *wR* and goodness of fit *S* are based on *F*^2^, conventional *R*-factors *R* are based on *F*, with *F* set to zero for negative *F*^2^. The threshold expression of *F*^2^ > σ(*F*^2^) is used only for calculating *R*-factors(gt) *etc*. and is not relevant to the choice of reflections for refinement. *R*-factors based on *F*^2^ are statistically about twice as large as those based on *F*, and *R*- factors based on ALL data will be even larger. EXTI refined to 0 and was removed from the refinement.

### Fractional atomic coordinates and isotropic or equivalent isotropic displacement parameters (Å^2^)

**Table:**

	*x*	*y*	*z*	*U*_iso_*/*U*_eq_	
Zr	0.628126 (8)	0.214931 (8)	0.623919 (7)	0.02366 (4)	
N1	0.62324 (8)	0.32650 (7)	0.59830 (7)	0.0296 (3)	
N2	0.67984 (8)	0.15023 (7)	0.54191 (7)	0.0302 (3)	
N3	0.68857 (8)	0.19705 (7)	0.72277 (7)	0.0293 (3)	
N4	0.78205 (8)	0.25715 (8)	0.60724 (7)	0.0294 (3)	
Si1	0.55541 (3)	0.39089 (2)	0.63723 (2)	0.02935 (9)	
Si2	0.63047 (3)	0.11014 (2)	0.46778 (2)	0.02709 (9)	
Si3	0.66444 (3)	0.12547 (3)	0.78173 (3)	0.03368 (10)	
C1	0.69404 (11)	0.35295 (10)	0.55145 (10)	0.0402 (4)	
H1A	0.6881	0.4068	0.5428	0.048*	
H1B	0.6922	0.3273	0.5039	0.048*	
C2	0.77345 (10)	0.13734 (10)	0.54823 (10)	0.0377 (4)	
H2A	0.7937	0.1066	0.5071	0.045*	
H2B	0.7864	0.1110	0.5942	0.045*	
C3	0.76119 (10)	0.24758 (10)	0.73870 (9)	0.0343 (3)	
H3A	0.7885	0.2335	0.7853	0.041*	
H3B	0.7402	0.2991	0.7430	0.041*	
C4	0.77852 (10)	0.33693 (9)	0.58941 (10)	0.0376 (4)	
H4A	0.8270	0.3503	0.5572	0.045*	
H4B	0.7830	0.3667	0.6345	0.045*	
C5	0.81823 (10)	0.21178 (10)	0.54734 (9)	0.0380 (4)	
H5A	0.8807	0.2051	0.5544	0.046*	
H5B	0.8088	0.2367	0.5000	0.046*	
C6	0.82595 (10)	0.24200 (10)	0.67714 (9)	0.0348 (4)	
H6A	0.8726	0.2783	0.6847	0.042*	
H6B	0.8514	0.1918	0.6762	0.042*	
C7	0.46795 (15)	0.41750 (14)	0.57399 (13)	0.0659 (7)	
H7A	0.4927	0.4371	0.5289	0.099*	
H7B	0.4321	0.4555	0.5968	0.099*	
H7C	0.4329	0.3739	0.5628	0.099*	
C8	0.61459 (15)	0.47570 (12)	0.66494 (16)	0.0670 (7)	
H8A	0.6359	0.5012	0.6215	0.101*	
H8B	0.6630	0.4619	0.6961	0.101*	
H8C	0.5760	0.5086	0.6918	0.101*	
C9	0.50645 (14)	0.35016 (11)	0.72057 (10)	0.0486 (5)	
H9A	0.4616	0.3149	0.7066	0.073*	
H9B	0.4813	0.3897	0.7502	0.073*	
H9C	0.5507	0.3245	0.7488	0.073*	
C10	0.69439 (13)	0.12628 (12)	0.38323 (9)	0.0491 (5)	
H10A	0.7510	0.1034	0.3885	0.074*	
H10B	0.7011	0.1796	0.3754	0.074*	
H10C	0.6646	0.1043	0.3415	0.074*	
C11	0.52293 (11)	0.15334 (11)	0.45402 (10)	0.0430 (4)	
H11A	0.4847	0.1388	0.4939	0.064*	
H11B	0.4988	0.1365	0.4076	0.064*	
H11C	0.5288	0.2073	0.4533	0.064*	
C12	0.62003 (13)	0.00839 (10)	0.48219 (12)	0.0458 (4)	
H12A	0.6771	−0.0134	0.4883	0.069*	
H12B	0.5917	−0.0140	0.4400	0.069*	
H12C	0.5858	−0.0009	0.5259	0.069*	
C13	0.76108 (14)	0.09490 (13)	0.83365 (13)	0.0587 (6)	
H13A	0.8074	0.0831	0.7996	0.088*	
H13B	0.7469	0.0508	0.8623	0.088*	
H13C	0.7796	0.1347	0.8663	0.088*	
C14	0.62506 (14)	0.04485 (11)	0.72743 (12)	0.0524 (5)	
H14A	0.6661	0.0338	0.6885	0.079*	
H14B	0.5692	0.0569	0.7061	0.079*	
H14C	0.6192	0.0016	0.7592	0.079*	
C15	0.58017 (14)	0.15179 (12)	0.84950 (11)	0.0493 (5)	
H15A	0.6006	0.1935	0.8788	0.074*	
H15B	0.5682	0.1095	0.8813	0.074*	
H15C	0.5277	0.1661	0.8238	0.074*	
C16	0.48882 (11)	0.17738 (10)	0.64014 (10)	0.0399 (4)	
H16A	0.4821	0.1268	0.6219	0.060*	
H16B	0.4504	0.2106	0.6134	0.060*	
H16C	0.4746	0.1788	0.6921	0.060*	

### Atomic displacement parameters (Å^2^)

**Table:**

	*U*^11^	*U*^22^	*U*^33^	*U*^12^	*U*^13^	*U*^23^
Zr	0.01895 (7)	0.02612 (7)	0.02593 (7)	−0.00195 (5)	0.00175 (5)	−0.00244 (5)
N1	0.0233 (6)	0.0322 (7)	0.0333 (6)	−0.0023 (5)	0.0047 (5)	0.0056 (5)
N2	0.0207 (6)	0.0368 (7)	0.0331 (7)	0.0001 (5)	−0.0010 (5)	−0.0091 (6)
N3	0.0277 (6)	0.0310 (6)	0.0293 (6)	−0.0040 (5)	−0.0004 (5)	0.0010 (5)
N4	0.0201 (6)	0.0367 (7)	0.0314 (6)	−0.0039 (5)	0.0022 (5)	−0.0038 (5)
Si1	0.0265 (2)	0.0259 (2)	0.0356 (2)	−0.00213 (16)	−0.00132 (17)	0.00063 (17)
Si2	0.02662 (19)	0.0296 (2)	0.02508 (19)	−0.00250 (16)	−0.00016 (16)	−0.00074 (16)
Si3	0.0355 (2)	0.0320 (2)	0.0335 (2)	0.00130 (18)	0.00282 (19)	0.00533 (18)
C1	0.0315 (8)	0.0440 (9)	0.0450 (10)	−0.0025 (7)	0.0093 (7)	0.0153 (8)
C2	0.0243 (7)	0.0475 (10)	0.0412 (9)	0.0053 (7)	−0.0011 (6)	−0.0145 (8)
C3	0.0352 (8)	0.0383 (8)	0.0295 (8)	−0.0074 (7)	−0.0037 (6)	−0.0029 (7)
C4	0.0264 (8)	0.0383 (9)	0.0482 (10)	−0.0090 (7)	0.0090 (7)	0.0055 (8)
C5	0.0206 (7)	0.0563 (11)	0.0372 (9)	−0.0022 (7)	0.0053 (6)	−0.0089 (8)
C6	0.0233 (7)	0.0430 (9)	0.0380 (9)	−0.0065 (7)	−0.0058 (6)	−0.0052 (7)
C7	0.0610 (14)	0.0767 (16)	0.0600 (13)	0.0338 (12)	−0.0199 (11)	−0.0094 (12)
C8	0.0627 (14)	0.0414 (11)	0.0969 (19)	−0.0193 (10)	0.0106 (13)	−0.0183 (12)
C9	0.0580 (12)	0.0409 (10)	0.0470 (11)	0.0056 (9)	0.0205 (9)	−0.0002 (8)
C10	0.0489 (11)	0.0676 (13)	0.0307 (9)	−0.0012 (10)	0.0073 (8)	0.0052 (9)
C11	0.0326 (9)	0.0530 (11)	0.0433 (10)	0.0031 (8)	−0.0063 (7)	0.0083 (8)
C12	0.0477 (11)	0.0303 (8)	0.0595 (12)	−0.0051 (8)	0.0075 (9)	−0.0054 (8)
C13	0.0523 (12)	0.0595 (13)	0.0644 (13)	0.0087 (10)	−0.0095 (10)	0.0215 (11)
C14	0.0650 (13)	0.0348 (9)	0.0573 (12)	−0.0100 (9)	0.0060 (10)	0.0016 (9)
C15	0.0521 (12)	0.0534 (11)	0.0425 (10)	0.0007 (9)	0.0142 (9)	0.0079 (9)
C16	0.0290 (8)	0.0441 (10)	0.0465 (10)	−0.0095 (7)	0.0061 (7)	−0.0078 (8)

### Geometric parameters (Å, °)

**Table:**

Zr—N2	2.0709 (12)	C5—H5A	0.9900
Zr—N1	2.0715 (13)	C5—H5B	0.9900
Zr—N3	2.0719 (13)	C6—H6A	0.9900
Zr—C16	2.2963 (16)	C6—H6B	0.9900
Zr—N4	2.5383 (12)	C7—H7A	0.9800
N1—C1	1.4796 (19)	C7—H7B	0.9800
N1—Si1	1.7278 (14)	C7—H7C	0.9800
N2—C2	1.4834 (19)	C8—H8A	0.9800
N2—Si2	1.7242 (13)	C8—H8B	0.9800
N3—C3	1.484 (2)	C8—H8C	0.9800
N3—Si3	1.7286 (13)	C9—H9A	0.9800
N4—C4	1.479 (2)	C9—H9B	0.9800
N4—C6	1.481 (2)	C9—H9C	0.9800
N4—C5	1.484 (2)	C10—H10A	0.9800
Si1—C7	1.856 (2)	C10—H10B	0.9800
Si1—C8	1.860 (2)	C10—H10C	0.9800
Si1—C9	1.8628 (18)	C11—H11A	0.9800
Si2—C12	1.8646 (18)	C11—H11B	0.9800
Si2—C11	1.8678 (17)	C11—H11C	0.9800
Si2—C10	1.8690 (18)	C12—H12A	0.9800
Si3—C13	1.868 (2)	C12—H12B	0.9800
Si3—C14	1.870 (2)	C12—H12C	0.9800
Si3—C15	1.8723 (19)	C13—H13A	0.9800
C1—C4	1.519 (2)	C13—H13B	0.9800
C1—H1A	0.9900	C13—H13C	0.9800
C1—H1B	0.9900	C14—H14A	0.9800
C2—C5	1.516 (2)	C14—H14B	0.9800
C2—H2A	0.9900	C14—H14C	0.9800
C2—H2B	0.9900	C15—H15A	0.9800
C3—C6	1.520 (2)	C15—H15B	0.9800
C3—H3A	0.9900	C15—H15C	0.9800
C3—H3B	0.9900	C16—H16A	0.9800
C4—H4A	0.9900	C16—H16B	0.9800
C4—H4B	0.9900	C16—H16C	0.9800
			
N2—Zr—N1	113.48 (5)	N4—C5—H5B	110.1
N2—Zr—N3	111.87 (5)	C2—C5—H5B	110.1
N1—Zr—N3	111.57 (5)	H5A—C5—H5B	108.4
N2—Zr—C16	107.24 (6)	N4—C6—C3	109.00 (13)
N1—Zr—C16	106.40 (6)	N4—C6—H6A	109.9
N3—Zr—C16	105.72 (6)	C3—C6—H6A	109.9
N2—Zr—N4	73.33 (5)	N4—C6—H6B	109.9
N1—Zr—N4	73.43 (5)	C3—C6—H6B	109.9
N3—Zr—N4	73.86 (5)	H6A—C6—H6B	108.3
C16—Zr—N4	179.41 (5)	Si1—C7—H7A	109.5
C1—N1—Si1	118.72 (11)	Si1—C7—H7B	109.5
C1—N1—Zr	114.78 (10)	H7A—C7—H7B	109.5
Si1—N1—Zr	125.71 (7)	Si1—C7—H7C	109.5
C2—N2—Si2	115.83 (10)	H7A—C7—H7C	109.5
C2—N2—Zr	114.53 (10)	H7B—C7—H7C	109.5
Si2—N2—Zr	129.64 (7)	Si1—C8—H8A	109.5
C3—N3—Si3	120.19 (10)	Si1—C8—H8B	109.5
C3—N3—Zr	115.11 (10)	H8A—C8—H8B	109.5
Si3—N3—Zr	124.54 (7)	Si1—C8—H8C	109.5
C4—N4—C6	112.92 (13)	H8A—C8—H8C	109.5
C4—N4—C5	112.84 (13)	H8B—C8—H8C	109.5
C6—N4—C5	111.41 (13)	Si1—C9—H9A	109.5
C4—N4—Zr	106.52 (9)	Si1—C9—H9B	109.5
C6—N4—Zr	106.10 (9)	H9A—C9—H9B	109.5
C5—N4—Zr	106.46 (9)	Si1—C9—H9C	109.5
N1—Si1—C7	111.45 (9)	H9A—C9—H9C	109.5
N1—Si1—C8	111.34 (9)	H9B—C9—H9C	109.5
C7—Si1—C8	108.85 (12)	Si2—C10—H10A	109.5
N1—Si1—C9	108.99 (8)	Si2—C10—H10B	109.5
C7—Si1—C9	108.40 (11)	H10A—C10—H10B	109.5
C8—Si1—C9	107.70 (11)	Si2—C10—H10C	109.5
N2—Si2—C12	109.94 (8)	H10A—C10—H10C	109.5
N2—Si2—C11	109.44 (8)	H10B—C10—H10C	109.5
C12—Si2—C11	110.65 (9)	Si2—C11—H11A	109.5
N2—Si2—C10	110.66 (8)	Si2—C11—H11B	109.5
C12—Si2—C10	108.57 (10)	H11A—C11—H11B	109.5
C11—Si2—C10	107.55 (9)	Si2—C11—H11C	109.5
N3—Si3—C13	111.45 (9)	H11A—C11—H11C	109.5
N3—Si3—C14	108.66 (8)	H11B—C11—H11C	109.5
C13—Si3—C14	107.91 (11)	Si2—C12—H12A	109.5
N3—Si3—C15	112.32 (8)	Si2—C12—H12B	109.5
C13—Si3—C15	107.59 (10)	H12A—C12—H12B	109.5
C14—Si3—C15	108.79 (10)	Si2—C12—H12C	109.5
N1—C1—C4	108.61 (13)	H12A—C12—H12C	109.5
N1—C1—H1A	110.0	H12B—C12—H12C	109.5
C4—C1—H1A	110.0	Si3—C13—H13A	109.5
N1—C1—H1B	110.0	Si3—C13—H13B	109.5
C4—C1—H1B	110.0	H13A—C13—H13B	109.5
H1A—C1—H1B	108.3	Si3—C13—H13C	109.5
N2—C2—C5	108.29 (14)	H13A—C13—H13C	109.5
N2—C2—H2A	110.0	H13B—C13—H13C	109.5
C5—C2—H2A	110.0	Si3—C14—H14A	109.5
N2—C2—H2B	110.0	Si3—C14—H14B	109.5
C5—C2—H2B	110.0	H14A—C14—H14B	109.5
H2A—C2—H2B	108.4	Si3—C14—H14C	109.5
N3—C3—C6	108.65 (13)	H14A—C14—H14C	109.5
N3—C3—H3A	110.0	H14B—C14—H14C	109.5
C6—C3—H3A	110.0	Si3—C15—H15A	109.5
N3—C3—H3B	110.0	Si3—C15—H15B	109.5
C6—C3—H3B	110.0	H15A—C15—H15B	109.5
H3A—C3—H3B	108.3	Si3—C15—H15C	109.5
N4—C4—C1	108.64 (13)	H15A—C15—H15C	109.5
N4—C4—H4A	110.0	H15B—C15—H15C	109.5
C1—C4—H4A	110.0	Zr—C16—H16A	109.5
N4—C4—H4B	110.0	Zr—C16—H16B	109.5
C1—C4—H4B	110.0	H16A—C16—H16B	109.5
H4A—C4—H4B	108.3	Zr—C16—H16C	109.5
N4—C5—C2	107.87 (13)	H16A—C16—H16C	109.5
N4—C5—H5A	110.1	H16B—C16—H16C	109.5
C2—C5—H5A	110.1		
			
N2—Zr—N1—C1	−32.96 (13)	C1—N1—Si1—C8	−37.01 (16)
N3—Zr—N1—C1	94.52 (12)	Zr—N1—Si1—C8	132.28 (12)
C16—Zr—N1—C1	−150.64 (12)	C1—N1—Si1—C9	−155.67 (13)
N4—Zr—N1—C1	29.95 (11)	Zr—N1—Si1—C9	13.62 (12)
N2—Zr—N1—Si1	157.39 (8)	C2—N2—Si2—C12	74.34 (14)
N3—Zr—N1—Si1	−75.14 (10)	Zr—N2—Si2—C12	−106.18 (11)
C16—Zr—N1—Si1	39.70 (10)	C2—N2—Si2—C11	−163.93 (12)
N4—Zr—N1—Si1	−139.70 (10)	Zr—N2—Si2—C11	15.55 (12)
N1—Zr—N2—C2	92.84 (12)	C2—N2—Si2—C10	−45.59 (15)
N3—Zr—N2—C2	−34.49 (13)	Zr—N2—Si2—C10	133.89 (11)
C16—Zr—N2—C2	−149.97 (12)	C3—N3—Si3—C13	−26.69 (15)
N4—Zr—N2—C2	29.87 (11)	Zr—N3—Si3—C13	148.55 (10)
N1—Zr—N2—Si2	−86.65 (10)	C3—N3—Si3—C14	−145.46 (13)
N3—Zr—N2—Si2	146.03 (9)	Zr—N3—Si3—C14	29.77 (12)
C16—Zr—N2—Si2	30.54 (12)	C3—N3—Si3—C15	94.13 (14)
N4—Zr—N2—Si2	−149.62 (11)	Zr—N3—Si3—C15	−90.64 (11)
N2—Zr—N3—C3	92.41 (11)	Si1—N1—C1—C4	112.38 (14)
N1—Zr—N3—C3	−35.93 (12)	Zr—N1—C1—C4	−58.05 (17)
C16—Zr—N3—C3	−151.18 (11)	Si2—N2—C2—C5	120.48 (13)
N4—Zr—N3—C3	28.37 (10)	Zr—N2—C2—C5	−59.08 (16)
N2—Zr—N3—Si3	−83.04 (9)	Si3—N3—C3—C6	119.29 (13)
N1—Zr—N3—Si3	148.62 (8)	Zr—N3—C3—C6	−56.37 (16)
C16—Zr—N3—Si3	33.37 (10)	C6—N4—C4—C1	−144.45 (14)
N4—Zr—N3—Si3	−147.08 (9)	C5—N4—C4—C1	88.09 (15)
N2—Zr—N4—C4	121.87 (11)	Zr—N4—C4—C1	−28.38 (15)
N1—Zr—N4—C4	0.34 (10)	N1—C1—C4—N4	54.78 (18)
N3—Zr—N4—C4	−118.70 (11)	C4—N4—C5—C2	−146.42 (14)
N2—Zr—N4—C6	−117.57 (11)	C6—N4—C5—C2	85.33 (15)
N1—Zr—N4—C6	120.91 (11)	Zr—N4—C5—C2	−29.91 (15)
N3—Zr—N4—C6	1.87 (10)	N2—C2—C5—N4	56.44 (17)
N2—Zr—N4—C5	1.21 (10)	C4—N4—C6—C3	86.62 (16)
N1—Zr—N4—C5	−120.31 (11)	C5—N4—C6—C3	−145.17 (14)
N3—Zr—N4—C5	120.65 (11)	Zr—N4—C6—C3	−29.71 (15)
C1—N1—Si1—C7	84.74 (16)	N3—C3—C6—N4	54.96 (18)
Zr—N1—Si1—C7	−105.97 (12)		
